# Editorial: New insights in the health benefits of art

**DOI:** 10.3389/fmed.2024.1426187

**Published:** 2024-05-31

**Authors:** Olivier Beauchet, Andy Hau Yan Ho, Ian Koebner, Auriane Gros

**Affiliations:** ^1^Department of Medicine, University of Montreal and Research Centre of the Geriatric University Institute of Montreal, Montreal, QC, Canada; ^2^Department of Medicine, Division of Geriatric Medicine, Sir Mortimer B. Davis Jewish General Hospital and Lady Davis Institute for Medical Research, McGill University, Montreal, QC, Canada; ^3^Action Research for Community Health (ARCH) Laboratory, Psychology Program, School of Social Sciences, Nanyang Technological University, Singapore, Singapore; ^4^Lee Kong Chian School of Medicine, Nanyang Technological University, Singapore, Singapore; ^5^Palliative Care Centre for Excellence in Research and Education, Singapore, Singapore; ^6^Department of Romance Languages and Literatures—Research Affiliate, Cultural Agents Initiative, Harvard University, Cambridge, MA, United States; ^7^Centre Hospitalier Universitaire de Nice, Clinique Gériatrique du Cerveau et du Mouvement, Nice, France; ^8^Laboratoire CoBTeK, Université Côte d'Azur, Nice, France; ^9^UFR Médecine de Nice, Département d'Orthophonie, Nice, France

**Keywords:** art, health, benefits, prevention, promotion

## Asking the right questions

The past decade has been characterized by an increase in research on the health benefits of art, as well as a growing incorporation of arts-based activities into the health systems of several high-income countries (1). A scoping review by the World Health Organization (WHO) showed that engaging with art may enhance mental, physical and social health, across a wide range of art forms (e.g., music, dance, visual art) and target populations (e.g., from patients to individuals free of disease and from pediatrics to geriatrics) (2). Empirical evidence now clearly supports the hypothesis that arts-based activities have promising health benefits, from health promotion to disease prevention, but there are still many areas to explore and actions to take.

One area that requires more attention and effort according to the WHO scoping review is robust experimental data on the health benefits of art, especially those generated through randomized controlled trials (1). Other important domains of knowledge that call for more investigations include those concerning engagement variability in arts-based activities, the social benefit of art and digital art therapy, as well as those related to targeted populations such as older community dwellers. In addition, more information is needed on the mechanisms underlying the health benefits of art, and how to implement an arts-based activity for health purposes in practice and across cultural contexts.

With this in mind, we decided to propose a Research Topic on “*New insights into the health benefits of art*”. The aim was to better understand the mechanisms of art's health benefits, their fields of application from health promotion to prevention, and the criteria for successful implementation of arts-based activities in daily practice. We suggested that addressing these enduring questions may help to support the development of health policies that integrate the arts into ongoing efforts to improve global health.

## What effects do arts-based activities have on us?

Within this Research Topic, we reported on five randomized controlled trials (RCT), performed in Asian (i.e., Japan and Singapore), European (i.e., France), and North American (i.e., Canada and United States) countries. These studies confirm the health benefits of arts-based activities. Most of these RCTs (i.e., 60%) examined arts-based activities carried out in museums. The results show an enhancement of wellbeing and quality of life, as well as improvement of physical health. One key finding was a reduction in frailty, which is a state characterized by vulnerability to stressors due to decreased physiological reserves, among older community dwellers. Another RCT demonstrated an increase in resilience, which is a key component of quality of life for individuals living with chronic diseases (3).

Digital art therapy is a new model of care that offers a contemporary and dynamic approach to therapeutic intervention, leveraging digital technologies to enhance accessibility, engagement, creativity, and effectiveness in addressing mental health and wellbeing in today's digital age (4). This Research Topic includes the first study to provide a comprehensive process evaluation of an evidence-based digital integrative arts therapy program. Furthermore, a second study found that arts-based activities practiced in a hybrid format (i.e., in-person and online) significantly improved the mental health of older community dwellers compared to their controls. The results of these two studies are promising and helped establish the feasibility and validity of digital models for arts-based health activities.

## How do arts-based activities improve health?

Multiple benefits of arts-based activities have been reported over the past decade (2). In contrast, fewer studies have examined the explanatory mechanisms of these health benefits. This Research Topic helps to address this gap in the literature through the publication of several mechanistic studies. First, the role of emotion has been highlighted. One study describes the development of the role of positive emotion processing in art therapy. The relationship between emotion and arts-based activities is complex, as art has the power to evoke, express, and reflect emotions in various ways (5, 6). Through art, individuals can experience and understand the emotions of others, fostering empathy and compassion. Arts-based activities may also be analyzed as an aesthetic experience (6). This experience refers to the subjective encounter with beauty or art that stimulates one's senses, emotions, and cognition in a profound and meaningful way. It involves perceiving and appreciating the qualities, forms, or expressions of objects, ideas, or experiences that evoke a sense of pleasure. Second, the mechanism of art's health benefits may be examined by studying measures of stress in humans including salivary cortisol levels, skin conductance or heart rate (7). On this Research Topic, we reported the effects of a museum-based art activity on heart rate used as a proxy measure of autonomic nervous system functioning that regulates our fight-or-flight and rest-and-digest responses. The study demonstrated that this art-based activity significantly decreased full-day heart rate, suggesting a health benefit in participants. Third, cerebral activity may elucidate the health benefits of art. A study on this subject found that a museum visit increased activation in the left ventrolateral prefrontal region of the brain and that this activation was associated with reduced anxiety and stress. This result suggests that the level of engagement of attention processes while practicing arts-based activities may play a key role their health benefits. Fourth, another study examined the mechanisms by which arts-based activities can create health benefits, as well as the associated lived experiences. It refers to the active involvement or participation of individuals with art, whether it be through creation, appreciation, interpretation, or interaction. This concept encompasses a wide range of activities and experiences that involve engaging with art in various forms, including visual arts, performing arts, literature, music, film, and more (8). Such engagement is not only a means of personal expression and enjoyment but also a way to connect with others, explore different perspectives, and enrich one's understanding of the world. It fosters creativity, critical thinking, empathy, and cultural appreciation, making it an essential aspect of individual and societal wellbeing. This systematic review showed that engagement with the arts reduced cognitive decline and improved mental health in a healthy population.

## What should the next step be?

The development of arts-based health interventions is an evolving field with much potential for growth and innovation. Several next steps for advancing its development are needed. First, as the arts and health community of practice grows, it should be mindful of the need to adapt arts-based interventions to be culturally responsive. For instance, using the modification of a singing intervention for post-partum depression (PPD) as a case study, Warran et al.'s brief research report on this topic underscored the importance of cultural sensitivity and local stakeholder involvement in achieving successful implementation of arts-based health interventions as they move across cultural contexts. Second, we need more research to better understand the mechanisms of art on various health conditions. Third, we need to advocate for the integration of arts-based interventions into different healthcare settings from hospitals to residences for older adults. Collaboration with healthcare professionals in the development and implementation of art programs may complement existing treatments and support holistic approaches from patient care to health promotion in the general population. Fourth, expanding access to arts-based interventions in community settings such as schools, community centers, libraries, and cultural institutions is essential for scale. Developing partnerships with these local organizations may be helpful to develop outreach programs that promote creativity, self-expression, and mental wellness among diverse populations. Fifth, providing training and education for healthcare professionals, therapists, educators, and artists on the principles and modalities of arts-based interventions is essential to ensure accessibility and scalability toward integrating the arts into professional healthcare and social practices. Sixth, we need to explore the use of technology and digital platforms to enhance the delivery and accessibility of arts-based interventions. Developing digital tools, mobile apps, virtual reality experiences and online platforms may facilitate creative expression, engagement, and collaboration in therapeutic and wellness contexts. Seventh, cross-disciplinary collaboration through a living lab approach is required to improve the effectiveness and personalization of arts-based interventions, as well as their implementation in health systems. Indeed, we need to create opportunities for knowledge exchange, cross-pollination of ideas, and co-creation of innovative solutions that leverage the intersection of art, health and technology. Eighth, we should advocate for policies and funding initiatives that support the integration of arts-based interventions into healthcare, education, and social services. There is a need to raise awareness about the health benefits of art and advocate for greater recognition and investment in arts-based approaches to health promotion, prevention, and treatment. By advancing these efforts, we can further harness the transformative power of art to improve health outcomes, enhance quality of life, and foster wellbeing for individuals and communities around the world.

## Author contributions

OB: Writing – original draft, Writing – review & editing. AH: Writing – review & editing. IK: Writing – review & editing. AG: Writing – review & editing.
